# NEDD8-conjugating enzyme UBC12 as a novel therapeutic target in esophageal squamous cell carcinoma

**DOI:** 10.1038/s41392-020-00226-3

**Published:** 2020-07-10

**Authors:** Shiwen Wang, Jingrong Xian, Lihui Li, Yanyu Jiang, Yue Liu, Lili Cai, Robert M. Hoffman, Lijun Jia, Hu Zhao, Yanmei Zhang

**Affiliations:** 1 Department of Laboratory Medicine, Huadong Hospital Affiliated to Fudan University, Shanghai, 200040 China; 2 Cancer Institute, Longhua Hospital, Shanghai University of Traditional Chinese Medicine, Shanghai, 200032 China; 3 Research Center on Aging and Medicine, Fudan University, Shanghai, 200040 China; 4 Shanghai Key Laboratory of Clinical Geriatric Medicine, Shanghai, 200040 China; 5 Department of Surgery, University of California, San Diego, CA USA

**Keywords:** Target identification, Gastrointestinal cancer

**Dear Editor**,

Esophageal squamous cell carcinoma (ESCC) is the major histologic subtype of esophageal cancer with high incidence and mortality.^1^ However, few achievements have been made in the development of targeted drugs.^2^ Neddylation, a reversible post-translational modification, attaches ubiquitin-like molecule NEDD8 to substrates in a three-step enzymatic reaction catalyzed by NEDD8-activating enzyme E1 (NAE, NAE1 and UBA3 heterodimer), NEDD8-conjugating enzyme E2s (UBE2M/UBC12 or UBE2F) and substrate-specific NEDD8-E3 ligases.^3^ The best-characterized substrates of neddylation are cullin family proteins, the essential components of multiunit Cullin-RING ubiquitin ligases (CRLs).^4^ Currently, the inhibition of cullin neddylation by targeting overactivated neddylation pathway has emerged as an attractive approach for anticancer therapy.^5,6^ Our previous study reported that MLN4924, a specific inhibitor of NAE, significantly inhibited the tumor growth of ESCC by blocking cullin neddylation and inactivating CRLs activity.^7^ However, recent studies found that MLN4924 treatment-emergent NAE mutations would confer the drug resistance.^8,9^ Therefore, it is urgent to identify other neddylation enzymes (E2s or E3s) as alternative anticancer targets and develop novel anti-ESCC strategies.

In the present study, with a label-free quantitative proteomic approach, NEDD8-conjugating enzyme UBC12 was identified as a potential anticancer target against ESCC. Gene ontology (GO) analysis of proteins with altered expression revealed that silencing UBC12 by CRISPR/Cas9 system significantly triggered a series of tumor-suppressive cellular responses of ESCC cells, as indicated by the up-regulated proteins involved in the regulation of apoptotic process, positive regulation of programmed cell death, cellular response to DNA damage stimulus, negative regulation of cell cycle process and negative regulation of growth (Fig. 1a), and the down-regulated proteins involved in the regulation of microtubule cytoskeleton organization, positive regulation of cell cycle, negative regulation of apoptotic process, positive regulation of cell growth and protein neddylation (Supplementary Fig. S1). These findings indicated that downregulation of UBC12 activated a series of tumor-suppressive cellular responses, providing the rationality for further evaluation of UBC12 as a potential anti-ESCC target.

**Fig. 1:**
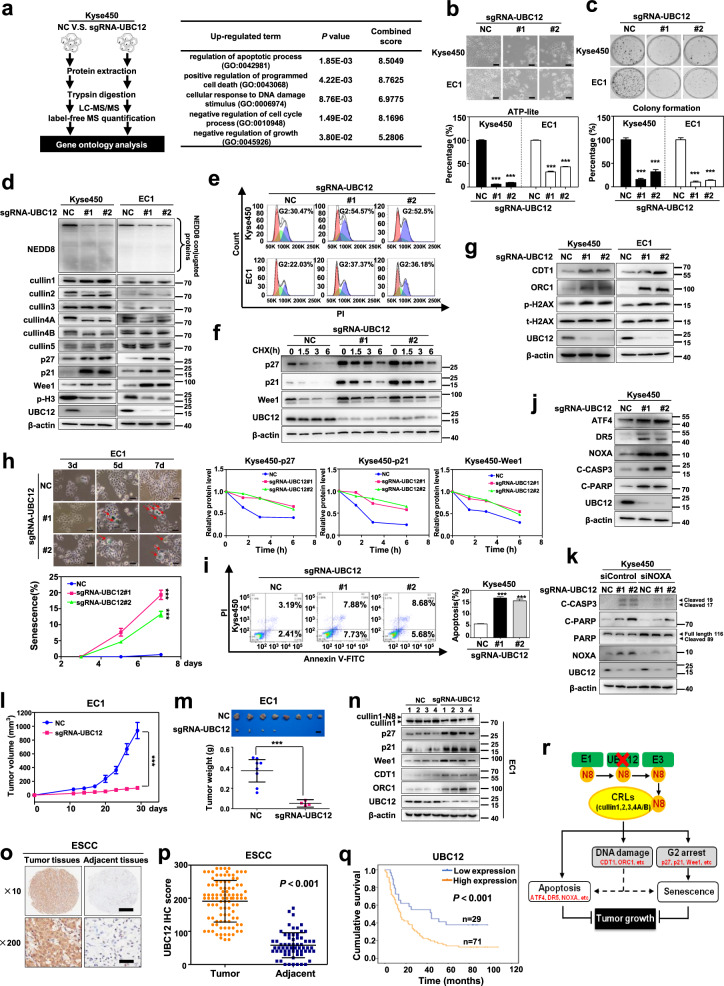
Validation of UBC12 as a new anticancer target in ESCC. **a** GO analysis based on quantitative proteomics strategy was used to reveal the changed cellular responses upon UBC12 knockdown. **b** UBC12-knockdown Kyse450 and EC1 stable cells with two different sgRNA-UBC12 oligos were generated by CRISPR/Cas9 system, and subjected to the micrograph and cell proliferation analysis using ATPlite assay. Scale bar = 200 μm. **c** The colony forming ability of UBC12-knockdown ESCC cells was determined by cell colony formation assay. **d** Immunoblotting was used to analyze the neddylation levels of global protein, cullin 1, 2, 3, 4A, 4B, and 5, as well as p27, p21, Wee1 and p-H3 upon UBC12 knockdown with β-actin as a loading control. **e** PI staining and FACS analysis were used to analyze the cell cycle profile upon UBC12 knockdown. **f** CHX was used to block protein synthesis, and protein lysates were extracted and subjected to immunoblotting against p27, p21, and Wee1 with β-actin as a loading control. **g** Immunoblotting analysis was used to assess the expression levels of CDT1, ORC1 and phosphorylated/total H2AX upon UBC12 knockdown with β-actin as a loading control. **h** Senescent EC1 cells with positive β-Galactosidase staining were pointed out with arrows. Scale bar = 50 μm. Statistical analysis showed that UBC12 knockdown time-dependently induced EC1 cell senescence. **i** UBC12 knockdown induced apoptosis of Kyse450 cells determined by AnnexinV-FITC/ PI double-staining analysis. **j** Immunoblotting analysis was used to assess the expression levels of apoptotic related proteins ATF4, DR5, NOXA, cleaved PARP and cleaved caspase3 upon UBC12 knockdown in Kyse450 cells. **k** Stable cells were transfected with either siControl or siNOXA for 72 h, and then subjected to immunoblotting analysis for cleaved caspase3, cleaved PARP, PARP and NOXA with β-actin as a loading control. **l** Tumor size was determined by caliper measurement, and the data were converted to tumor growth curve. **m** Mice were sacrificed and tumor tissues were harvested and photographed. Scale bar = 1 cm. The tumor weight was obtained on the sacrificed day. **n** Proteins extracted from tumor tissues and subjected to immunoblotting analysis against cullin 1, p27, p21, Wee1, CDT1, ORC1 and UBC12 with β-actin as a loading control. **o** IHC staining of human ESCC tissue arrays using specific antibody for UBC12. Scale bar for ×10 images, 500 μm; Scale bar for ×200 images, 25 μm. **p** The expression difference of UBC12 in the ESCC tissues of patients and adjacent normal tissues by histological evaluation. **q** Kaplan–Meier curves for overall survival rate of patients with ESCC based on UBC12 expression. **r** Shown was the schema of the mechanism for UBC12 knockdown suppressing tumor growth in ESCC. Representative images were shown. Shown were average values with standard deviation. *** denotes the *P* < 0.001.

To verify the above mass spectra findings, we first systematically evaluated the effects of UBC12 knockdown on malignant phenotypes of ESCC cells. We found that UBC12 knockdown dramatically inhibited the cell proliferation, colony formation (Fig. 1b, c), as well as the transwell migration and invasion abilities of ESCC cells (Supplementary Fig. S2a–d). In mechanisms, the level of global protein neddylation was remarkably suppressed upon UBC12 knockdown. Moreover, UBC12 downregulation dramatically decreased the neddylation levels of cullin 1, 2, 3, 4A, and 4B, the substrates of UBC12, but not cullin 5, the substrate of UBE2F (Fig. 1d), that such, resulting in the inactivation of CRLs and the corresponding induction of abnormal cellular responses. GO analysis results of proteins with altered expression suggested that the cell cycle procession was remarkably disturbed upon UBC12 knockdown (Fig. 1a and Supplementary Fig. S1), as confirmed by the upregulation of cell cycle inhibitors p27, p21 and Wee1, and the downregulation of M phase marker phosphorylated-histone H3 (Fig. 1d). Consistently, cell cycle profile analysis proved that the cell populations in G2 phase were significantly increased in both two UBC12-knockdown ESCC cell lines (Fig. 1e). Since p27, p21 and Wee1 also served as the substrates of CRLs, in turn, the half-lives of these cell cycle inhibitors were found to be dramatically extended owing to CRLs inactivation when silencing UBC12 (Fig. 1f). Therefore, UBC12 downregulation effectively blocked cullin neddylation to inactivate CRLs, leading to G2 phase cell cycle arrest in ESCC cells.

Consistent with aforementioned mass spectra findings, we showed that CRLs substrates CDT1 and ORC1, two DNA replication licensing proteins, were obviously accumulated upon UBC12 knockdown, and subsequently led to DNA damage response, as reflected by increased levels of phosphorylated H2AX (Fig. 1g). Further phenotypic analysis revealed that UBC12 knockdown triggered senescence or apoptosis of ESCC cells in a cell line-dependent manner (Fig. 1h, i). UBC12-knockdown EC1 cells displayed classical senescence morphology with an enlarged and flattened cellular shape and the positive staining for senescence-associated β-Galactosidase (Fig. 1h). Unlike, the notable apoptosis feature of shrunk morphology was presented in UBC12-knockdown Kyse450 cells, which was accompanied with the significant increase of Annexin V-positive cells (Fig. 1i). Furthermore, we found that UBC12 knockdown induced the accumulation of CRLs substrate activating transcription factor 4 (ATF4), and therefore transactivated death receptor 5 (DR5) to trigger extrinsic apoptosis of Kyse450 cells. On the other hand, the apoptotic protein NOXA, another downstream target of ATF4, was also activated and triggered the intrinsic apoptosis (Fig. 1j). Additionally, NOXA downregulation via siRNA silencing significantly suppressed apoptotic induction induced by UBC12 downregulation (Fig. 1k). Together, these results suggested that UBC12 downregulation significantly diminished the growth of Kyse450 cells through activation of both extrinsic apoptosis and intrinsic apoptosis pathways.

To investigate the therapeutic potential of UBC12 silencing in vivo, we established the subcutaneous-transplantation tumor model using EC1 cells. We found that downregulation of UBC12 significantly inhibited tumor growth, as analyzed by tumor growth curve (*P* < 0.001, Fig. 1l) and tumor weight (*P* < 0.001, Fig. 1m). Mechanistic studies revealed that silencing UBC12 led to cullin neddylation inhibition and CRLs inactivation. As a result, CRLs substrates p27, p21, Wee1, CDT1 and ORC1 were obviously accumulated (Fig. 1n). These findings were further supported by immunohistochemical staining (IHC) analysis, which showed fewer Ki67 positive cells and more p27 positive cells in UBC12-knockdown group than control group (Supplementary Fig. S3a, b).

To further evaluate the activation status of UBC12 in ESCC, we determined the UBC12 expression by IHC staining using tissue arrays derived from human ESCC. We observed that UBC12 were overexpressed in the ESCC tissues compared with adjacent normal tissues (Fig. 1o). The results of histologic evaluation showed that the UBC12 expression levels were significantly elevated in ESCC tissues of patients (*P* < 0.001, Fig. 1p). Importantly, Kaplan-Meier analysis indicated that the overall survival rate was lower in ESCC patients with high expression of UBC12 than in patients with low expression (*P* < 0.001, log-rank test, Fig. 1q and Supplementary Table S1).

Collectively, our work validated UBC12 as a promising anticancer target against ESCC with the following evidences (Fig. 1r): (a) Mass spectra analysis showed that UBC12 silencing induced a series of tumor-suppressive cellular responses; (b) UBC12 knockdown profoundly inhibited the malignant phenotypes of ESCC cells both in vitro and in vivo; (c) Mechanistically, downregulation of UBC12 inactivated CRLs and induced the accumulation of CRLs substrates (e.g. p27 and p21), therefore triggering DNA damage, cell cycle arrest, senescence and/or apoptosis; (d) Finally, UBC12 was overexpressed in ESCC tissues and predicted a poor overall survival of patients. These findings provide additional choices for targeting the overactivated neddylation pathway for anti-ESCC therapy. Given the fact that UBE2F has also been reported as a potential lung cancer target and biomarker.^10^ Therefore, in combination with our findings, targeting these two neddylation E2s: UBE2M and UBE2F will be a promising approach for anticancer therapy. The development of UBE2M and UBE2F inhibitors may overcome emerging resistance to neddylation E1 inhibitors (eg, MLN4924) as a result of treatment-related mutations.

## Supplementary information

Supplementary information
