# Supplementary material for: NEDD8-conjugating enzyme UBC12 as a novel therapeutic target in esophageal squamous cell carcinoma
Source: Signal Transduct Target Ther. 2020 Jul 10;5:123. doi: 10.1038/s41392-020-00226-3 (PMC7351728; doi:10.1038/s41392-020-00226-3)
Supplement: Supplementary file 1 — Supplementary information [file 41392_2020_226_MOESM1_ESM.pdf]

Supplementary Materials for

NEDD8-conjugating enzyme UBC12 as a novel therapeutic target in esophageal squamous cell carcinoma

Shiwen Wang[1,2,3,4†], Jingrong Xian[1,2,3,4†], Lihui Li[2], Yanyu Jiang[2], Yue Liu[1,2,3,4], Lili Cai[2], Robert M. Hoffman[5], Lijun Jia[2*], Hu Zhao[1,3,4*], Yanmei Zhang[1,3,4*]

[1] Department of Laboratory Medicine, Huadong Hospital Affiliated to Fudan University, Shanghai, 200040, China
[2] Cancer Institute, Longhua Hospital, Shanghai University of Traditional Chinese Medicine, Shanghai, 200032, China
[3] Research Center on Aging and Medicine, Fudan University, Shanghai, 200040, China
[4] Shanghai Key Laboratory of Clinical Geriatric Medicine, Shanghai, 200040, China
[5] Department of Surgery, University of California, San Diego, CA, USA
[†] These authors contributed equally to this work.

*Correspondence e-mails: 15618653286@163.com to Yanmei Zhang
hubertzhao@163.com to Hu Zhao
jialijun2002@aliyun.com to Lijun Jia

This file includes:

Supplemental Figures
Fig. S1 UBC12 knockdown induces tumor-suppressive cellular responses of ESCC.
Fig. S2 UBC12 knockdown suppresses the transwell migration and invasion of ESCC cells.
Fig. S3 UBC12 knockdown suppresses the tumor growth of ESCC analyzed by IHC staining.
Supplemental Table
Table S1 Clinicopathologic parameters according to the expression of UBC12.
Materials and Methods
References

Down-regulated term	*P* value	Combined score
regulation of microtubule cytoskeleton organization (GO:0070507)	1.64E-03	28.7798
positive regulation of cell cycle (GO:0045787)	8.46E-03	10.8754
negative regulation of apoptotic process (GO:0043066)	1.71E-02	5.7529
positive regulation of cell growth (GO:0030307)	2.87E-02	6.7835
protein neddylation (GO:0045116)	3.61E-02	13.1721

Fig. S1 UBC12 knockdown induces tumor-suppressive cellular responses of ESCC. GO analysis based on quantitative proteomics strategy was used to reveal the changed cellular responses upon UBC12 knockdown.

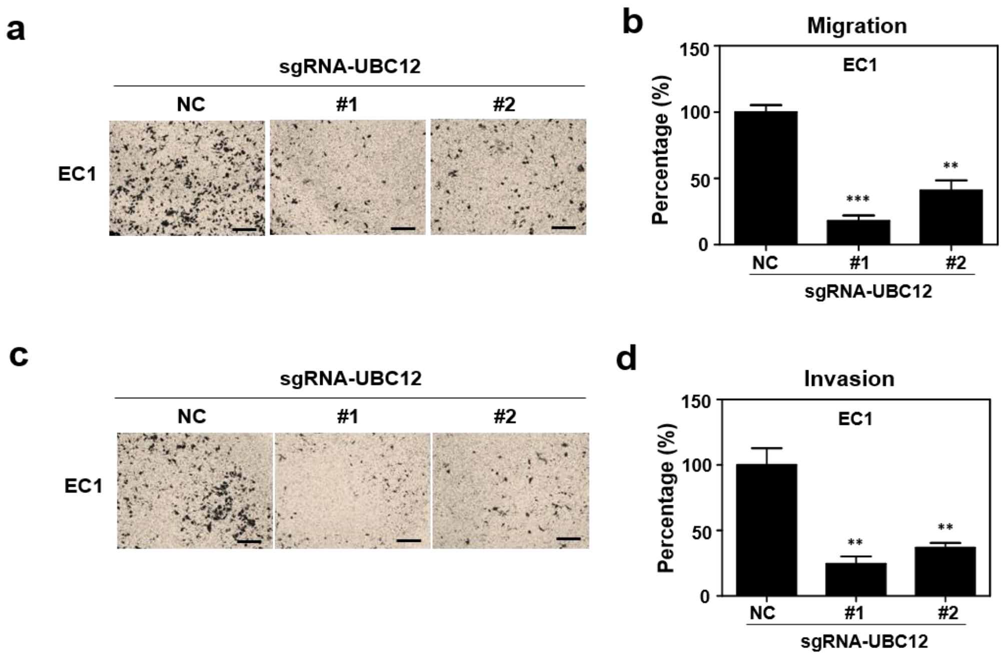

Fig. S2 UBC12 knockdown suppresses the transwell migration and invasion of ESCC cells. UBC12-knockdown EC1 stable cells with two different sgRNA-UBC12 oligos were generated by CRISPR/Cas9 system. **a, b** The migration ability of UBC12-knockdown EC1 cells was determined by transwell migration assay and compared with that of the control group. **c, d** The invasion ability of UBC12-knockdown EC1 cells was determined by transwell invasion assay and compared with that of the control group. Representative images were shown. Scale bar = 200μm. Shown were average values with standard deviation. **denotes the $P < 0.01$ and ***denotes the $P < 0.001$.

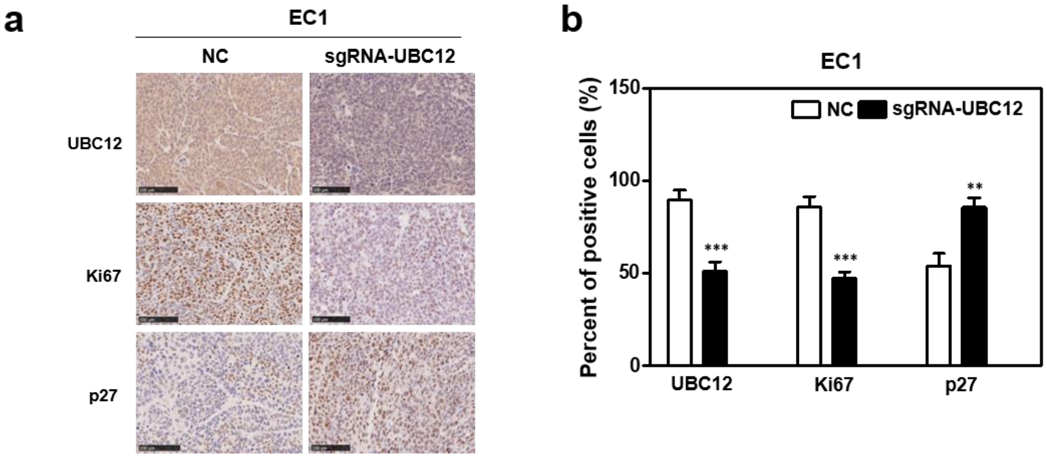

Fig. S3 UBC12 knockdown suppresses the tumor growth of ESCC analyzed by IHC staining. a IHC staining of tumor tissues with specific antibodies for UBC12, Ki67 and p27. Representative images were shown. Scale bar = 100μm. **b** Statistical analysis was performed to analyze the percent of positive cells of UBC12, Ki67 and p27. Shown were average values with standard deviation. **denotes the $P < 0.01$ and ***denotes the $P < 0.001$.

Table S1 Clinicopathologic parameters according to the expression of UBC12

**Variable**	**Overall**	**UBC12**		***P***
	**No.**	**Low**	**High**	
**Age (n*=100)**				0.006
<60	26	13	13	
≥60	74	16	58	
**Gender (n*=100)**				0.082
male	74	18	56	
female	26	11	15	
**Tumor (n*=100)**				0.781
T1	5	1	4	
T2	16	6	10	
T3	78	22	56	
T4	1	0	1	
**Node (n*=100)**				0.983
N_0	48	15	33	
N_1	31	9	22	
N_2	16	4	12	
N_3	5	1	4	
**TNM (n*=100)**				0.354
Ⅰ	6	2	4	
Ⅱ	45	16	29	
Ⅲ	49	11	38	

Materials and Methods

Cell culture and reagents

Human ESCC cell lines Kyse450, and EC1 were obtained from the American Type Culture Collection (Manassas, VA) and cultured in Dulbecco's Modified Eagle's Medium (DMEM, Hyclone, Logan, UT), containing 10% fetal bovine serum (Biochrom AG, Berlin, Germany) and 1% penicillin-streptomycin solution at 37°C with 5% carbon dioxide.

Generation of UBC12-knockdown stable cell lines by CRISPR/Cas9 system

For packaging of lenti-virus in ESCC cells, two different sgRNA (small guide RNA) oligos against UBC12 were inserted into vector lenti-guide-puro (4.0 μg) respectively, which co-transfected with packaging plasmids AGP091 (3.0 μg) and AGP090 (1.2 μg) into 293T cells using Lipofectamine 2000 (Invitrogen, Carlsbad, CA, USA). Two human sgRNA oligos sequences for UBC12 were as follows: 5‘-TCACCAAGAAGAGATACTGC-3’ and 5‘-AGATGACCAGCTTGAAGTTG-3’; Forty-eight hours after transfection, the viral supernatant was harvested, filtered and then mixed with 10μg/mL Polybrene (sigma-Aldrich, St. louis, MO) to increase the infection efficiency. Kyse450 and EC1 cells were infected with the virus and selected in 1 μg/mL puromycin (Invitrogen, Carlsbad, CA) for 3 days.

LC-MS/MS analysis and label-free MS quantification

LC-MS/MS analysis and label-free based MS quantification for proteins were performed as previously described[1]. MS raw files were processed with the Firmiana proteomics workstation[2]. Briefly, the trypsin digested peptides from Kyse450 cells lyses were subjected to MS analysis on a Fusion Lumos (Thermo Fisher Scientific). Raw files were searched against the NCBI human Refseq protein database (released on 04-07-2013, 32,015 entries) in Mascot search engine (version 2.3, Matrix Science Inc.) with FDR < 1% at peptide and protein levels. The database searching considered cysteine carbamidomethylation as a fixed modification and N-acetylation, oxidation of methionine as variable modifications. For protein quantification, we used intensity-based label-free quantification, the so called iBAQ algorithm. iBAQ value were converted to FOT–iBAQ value of each protein divided by the sum of all iBAQ values of all proteins in the sample. The FOT values were then multiplied by 10^6 to obtain FOT numbers to make easy visualization of low abundant proteins.

siRNA silencing

To knock-down NOXA, stable cells were transfected with siRNA oligonucleotides using Lipofectamine 2000 (Invitrogen, Carlsbad, CA, USA). siRNA oligonucleotides were synthesized by GenePharma (Shanghai, China). The sequences of the siRNAs

were as follows: siNOXA: 5’-GGUGCACGUUUCAUCAAUUUGTT-3’; siControl: 5’-UUCUCCGAACGUGUCACGUTT-3’.

Cell proliferation and colony formation assay

Cells were seeded in 96-well plates with 1500 cells per well in triplicate and cultured for 72 hours. The cell proliferation was determined by an ATPlite luminescence assay (PerkinElmer, Norwalk, CT, USA) according to the manufacturer’s protocol. For colony formation assays, cells were seeded into 6-well plates with 300 cells per well in triplicate followed by incubating at 37 °C for 14 days. The colonies on the plates were fixed with 4% paraformaldehyde for 20 minutes and stained in dye solution containing 0.1% crystal violet for 30 minutes. Colonies with more than 50 cells were counted and photographed under an inverted microscope (Olympus, Tokyo, Japan). Representative results of three independent experiments with similar trends were presented.

Transwell migration and invasion assay

The migration and invasion of the cells was assayed using a transwell polycarbonate filter (8-μm pore size; Corning, Lowell, MA). Briefly, the top chambers were seeded in 24-well plates with 5×10^4 cells per well in triplicate in serum-free medium, and the bottom chambers were filled with 600 uL per chamber medium containing 10% FBS. For invasion assay, the suspension was placed into the top chamber with a Matrigel-coated polycarbonate membrane (Corning). After 24 hours incubation at 37°C, the top chambers were fixed in 4% paraformaldehyde for 20 minutes and stained with 0.1% crystal violet for 30 minutes. Cells that passed through the polycarbonate membrane were counted under an inverted microscope (Olympus, Tokyo, Japan). Representative results of three independent experiments with similar trends were presented.

Cell cycle profile analysis

Cells were harvested and fixed in 70% ethanol at -20°C overnight. Then, the fixed cells were stained with propidium iodide (PI, 36 µg/mL; Sigma, St. Louis, MO, USA) containing RNase A (10 µg/mL Sigma, St. Louis, MO, USA) at 37 °C for 15 min, and performed fluorescence-activated cell sorting (FACS) analysis by Flow Cytometry (BD FACSVerse™). Data were analyzed by Flowjo 7.6 software, and dean-jett-fox model (Dean, Jett 1974) was used to distinguish G1, S and G2 fractions.

Apoptosis assay

Cells were collected and stained with Annexin V-fluorescein isothiocyanate (FITC) and PI using an Annexin V-FITC Apoptosis Detection Kit (Beck-man Coulter)

according to the manufacturer's protocol, followed by flow cytometric analysis by flow cytometric (BD FACSVerse™). Data were analyzed by Flowjo 7.6 software.

SA-β-Galactosidase staining

For senescence detection, cells were seeded in 12-well plates with1×10^4 cells per well in triplicate and cultured for 72 hours. The expression of senescence-associated β-galactosidase was determined with the SA-β-galactosidase (SA-β-Gal) staining kit (Beyotime, Shanghai, China) according to the manufacturer's protocol.

Immunoblotting and cycloheximide (CHX)-chase assay

Cell lysates were prepared for immunoblotting analysis using the primary antibodies against UBC12, UBA3, cullin1, cullin2, cullin5, p21, NOXA (abcam), NAE1, cullin3, NEDD8, cullin4a, p27, Wee1, p-H3, p-H2AX, t-H2AX, ATF4, DR5, PARP, cleaved PARP, caspase3, cleaved caspase3 (Cell Signaling, Boston, MA), cullin4b (protein Tech) and β-actin (protein Tech). For CHX-chase assay, cells were treated with 50 μg/mL CHX (Sigma) at indicated time points and determined the half-lives of p27, p21 and Wee1 by immunoblotting analysis.

Immunohistochemistry and evaluation of human ESCC tissue arrays

Human ESCC tissue arrays were immunohistochemically stained with UBC12 antibody from Shanghai Outdo Biotech Co. Ltd. (Shanghai, China). Briefly, the tissue array sections were dehydrated and subjected to peroxidase blocking, following incubated with primary antibody at room temperature for 30 minutes. After staining with the GTVisionTM III Detection System/Mo&Rb (Gene tech Company Limited), the slides were counterstained with hematoxylin. The stained slides were observed under microscopy and acquired the images. For histologic evaluation, the tissue sections were quantitatively scored based on the percentage of positive tumor cells and the staining intensity as described previously[3,4]. Specifically, the mean percentage of positive tumor cells were calculated in five areas of a given sample at a magnification of ×200 and scored from 0 to 1 (0-100 %). The intensity of staining was scored as 0 for negative, 1 for weak, 2 for moderate, and 3 for strong. The proportion and intensity scores were then combined to obtain a total score for each case, ranging from 0 (0% of cells stained) to 300 (100% of cells stained at 3 intensity). We defined the score <150 as low expression and ≥150 as high expression. The detailed clinicopathologic characteristics of ESCC patients are presented in Supplementary Tables 1. The use of human ESCC tissues and the database was approved by the Research Ethics Committee of Taizhou Hospital.

Subcutaneous transplantation tumor model and immunohistochemistry

2×10^6 stable cells were subcutaneously injected into the back of the six-week BALB/c female nude mice. Tumor size was measured by a caliper and the tumor volume was calculated using the ellipsoid volume formula ($\text{length}\times\text{width}^2$)/2. Tumor tissues were harvested, photographed, and weighed, following fixed by 4% paraformaldehyde for immunohistochemical staining. In three random regions of tumor sections, UBC12/Ki67/p27 positive cells were quantitatively scored according to the histological evaluation, and the average value was taken to calculate the percentage of observed positive cells. Protein expression levels of the tumor tissues were evaluated by immunoblotting analysis using specific antibodies as indicated. Animal experiments were performed in accordance with animal protocols approved by the Institutional Animal Care and Use Committee of Fudan University.

Statistical analysis

Survival was calculated using the Kaplan-Meier analysis and compared with the log-rank test by Statistical Program for Social Sciences software (SPSS) Version 22.0. Data were presented as mean ± standard deviation. The statistical significance of differences between groups was assessed by GraphPad Prism5 software. (GraphPad Software, Inc., San Diego, CA, USA). The student's t-test was used for the comparison of parameters between two groups. $P < 0.05$ was considered statistically significant. For all tests, three levels of significance (***$P < 0.001$) were used.

References:

1 Ni, X.*et al*, A region-resolved mucosa proteome of the human stomach. *Nat. Commun.* **10**, 39 (2019).

2 Feng, J.*et al*, Firmiana: towards a one-stop proteomic cloud platform for data processing and analysis. *Nat. Biotechnol.* **35**, 409-412 (2017).

3 Zhou, L.*et al*, Promotion of tumor-associated macrophages infiltration by elevated neddylation pathway via NF-kappaB-CCL2 signaling in lung cancer. *Oncogene* **38**, 5792-5804 (2019).

4 Li, X.*et al*, A splicing switch from ketohexokinase-C to ketohexokinase-A drives hepatocellular carcinoma formation. *Nat. Cell Biol.* **18**, 561-571 (2016).
